# Prospective association between evening circadian preference and academic functioning in adolescents: the role of daytime sleepiness

**DOI:** 10.1111/jcpp.13683

**Published:** 2022-08-11

**Authors:** Joseph W. Fredrick, Taryn E. Cook, Joshua M. Langberg, Stephen P. Becker

**Affiliations:** ^1^ Division of Behavioral Medicine and Clinical Psychology Cincinnati Children's Hospital Medical Center Cincinnati OH USA; ^2^ Department of Pediatrics University of Cincinnati College of Medicine Cincinnati OH USA; ^3^ Department of Psychology Virginia Commonwealth University Richmond VA USA

**Keywords:** adolescence, circadian function, circadian preference, daytime sleepiness, academic impairment, ADHD, grades

## Abstract

**Background:**

There is growing evidence for the role of circadian factors in adolescents' sleep and academic adjustment, with greater evening preference being linked to poorer academic functioning. However, studies have yet to evaluate this association prospectively in adolescence, nor have studies examined daytime sleepiness as a putative mechanism linking evening preference to poor academic functioning. The current study used a multi‐informant design to test the prospective association of evening circadian preference, daytime sleepiness, and academic functioning (e.g., global academic impairment and grades) across 2 years in adolescence. As evening circadian preference, sleepiness, and academic problems are elevated in adolescents with ADHD, we used a sample enriched for adolescents with ADHD and explored whether ADHD moderated effects.

**Method:**

Participants were 302 adolescents (*M*_age_ = 13.17 years; 44.7% female; 81.8% White; 52% with ADHD). In the fall of eighth grade, adolescents reported on their circadian preference, and in the fall of ninth grade, adolescents and parents completed ratings of daytime sleepiness. In the middle of 10th grade, parents and teachers reported on adolescents' academic impairment and at the end of 10th grade, adolescents' grade point average (GPA) was obtained from school records.

**Results:**

Above and beyond covariates (e.g., adolescent sex, ADHD status, medication, sleep duration) and baseline academic impairment, greater self‐reported evening preference in 8th grade predicted increased parent ratings of academic impairment in 10th grade indirectly via adolescent and parent ratings of daytime sleepiness in 9th grade. Furthermore, evening preference in 8th grade predicted greater teacher ratings of academic impairment and lower average GPA in 10th grade via parent ratings of daytime sleepiness in 9th grade, controlling for covariates and baseline GPA. ADHD status did not moderate indirect effects.

**Conclusion:**

Findings underscore daytime sleepiness as a possible intervening mechanism linking evening preference to poor academic functioning across adolescence. Intervention studies are needed to evaluate whether targeting circadian preference and sleepiness improves academic functioning in adolescents.

## Introduction

There has been growing interest in the role of circadian factors in the development and maintenance of sleep and academic functioning in adolescence (Arns, Kooij, & Coogan, 2021), a developmental period characterized by substantial biological, psychological, and social processes impacting sleep–wake regulation (Becker, Langberg, & Byars, 2015; Crowley, Wolfson, Tarokh, & Carskadon, 2018). Circadian preference, often assessed via self‐report ratings, is characterized by the component of the sleep/wake cycle reflecting individuals' tendencies for the timing of waking activities and sleep (Jenni, Achermann, & Carskadon, 2005; Roeser, Schlarb, & Kübler, 2013). Circadian preference is considered a proxy for chronotype, which is assessed via biological indices (dim light melatonin onset), and research demonstrates significant associations between self‐reported ratings of circadian preference and biological indices of circadian function (Zavada, Gordijn, Beersma, Daan, & Roenneberg, 2005). Additionally, studies report moderate individual stability of self‐reported circadian preferences across adolescence (Kuula et al., 2018). Although adolescents undergo a natural biological shift toward later eveningness coinciding with pubertal development (Dolsen, Wyatt, & Harvey, 2019), adolescents vary on the morning–evening continuum. Specifically, adolescents with inclinations toward morning preference (‘morning lark’) generally prefer earlier sleep onset and waking activities, whereas those with an evening preference (‘night owl’) prefer later activities and sleep onset (Roeser et al., 2013). Across studies examining correlates of circadian preference, greater evening preference is linked to a host of emotional, social, sleep, and academic difficulties (Chen et al., 2021; Dolsen et al., 2019). Of particular concern, due to the lack of synchrony between later evening preference and expectations for early waking and school‐based activities, is the detrimental impact on academic functioning (Tonetti, Natale, & Randler, 2015). Studies have linked later evening preference to lower average school grades (Cohen‐Zion & Shiloh, 2018; Saxvig, Pallesen, Wilhelmsen‐Langeland, Molde, & Bjorvatn, 2012) and increased academic difficulties (Tonetti et al., 2015). Recently, a recent meta‐analysis of 23 studies reported a small yet significant association between greater evening preference and lower academic achievement (Scherrer & Preckel, 2021). However, the authors noted the absence of any longitudinal studies as a key limitation of extant research in this area.

## Need for longitudinal studies

Longitudinal studies spanning adolescence are critical for informing our understanding of whether evening circadian preference is directly related to academic outcomes, as well as whether other developmental processes that may be important for understanding this association. To our knowledge, only one study to date has explored the prospective association of circadian preference and academic impairments (Scherrer & Preckel, 2021). In a community‐based sample of adolescents, morning preference, but not evening preference, was associated with higher grade point average (GPA; Scherrer & Preckel, 2021) over a 2‐year period. However, the two time point design of the study precluded the opportunity to examine intervenining processes that may explain the link between evening circadian preference and academic outcomes.

One particularly compelling candidate mechanism is excessive daytime sleepiness (Sivertsen, Glozier, Harvey, & Hysing, 2015). Adolescents with later evening preference report shortened sleep duration, poorer sleep quality, and increased difficulties waking (Giannotti, Cortesi, Sebastiani, & Ottaviano, 2002; Roeser et al., 2013; Vollmer et al., 2017). Additionally, greater evening preference has been linked to delaying bedtime and sleep onset (Chung, An, & Suh, 2020). In turn, adolescents often experience increased drowsiness and poor sustained attention in the early hours of the school day (Roeser et al., 2013). Furthermore, evening preference may have a stronger prospective association with the development of daytime sleepiness compared to other sleep domains such as quality and duration (Luo, Zhang, Chen, Lu, & Pan, 2018). Regarding the association between daytime sleepiness and academic problems, studies have linked greater daytime sleepiness to poorer academic performance (Chung & Cheung, 2008) and lower school grades (Liu et al., 2016; Meijer, 2008). In fact, systematic reviews and meta‐analyses have observed stronger effects of daytime sleepiness on school performance compared to other types of sleep problems (Dewald, Meijer, Oort, Kerkhof, & Bögels, 2010; Tonetti et al., 2015). Thus, daytime sleepiness might be a key mechanism that explains the association of evening preference and academic outcomes in adolescence. Indeed, one cross‐sectional study found daytime sleepiness to mediate the link between evening preference and global GPA in adolescents (Sivertsen et al., 2015).

In addition, although daytime sleepiness is directly linked to poor academic performance (Dewald et al., 2010; Drake et al., 2003; Meijer, 2008), very few studies have tested individual factors associated in the development of daytime sleepiness (Luo et al., 2018) or evaluated *specific* academic domains. For instance, studies assessing evening preference and daytime sleepiness with academic outcomes often measure global ratings of academic impairment or collapse across subjects when assessing grades (Dewald et al., 2010; Scherrer & Preckel, 2021; Sivertsen et al., 2015). Thus, to improve our conceptualization of how evening preference may be associated with academic outcomes, this study examines parent and teacher ratings of academic impairment in addition to overall GPA obtained from school report cards. We also examined the effect of evening preference and daytime sleepiness on specific academic grades in supplemental analyses.

## Current study

This study builds on previous research by testing daytime sleepiness as an intervening mechanism linking evening circadian preference to academic impairments over 2 years in adolescence. Specifically, we tested whether self‐reported evening preference in 8th grade was related to academic outcomes in 10th grade *indirectly* via 9th grade daytime sleepiness. In addition to global ratings of parent‐ and teacher‐reported academic impairment, we collected school grades from report cards to provide a more specific and ecologically valid indicator of academic performance in adolescents (Scherrer & Preckel, 2021). As supplemental analyses given the absence of previous studies in this area, we also examined effects for specific academic subjects (i.e., english/language arts, history, math, science). Additionally, a multi‐informant approach using both self and parent ratings of daytime sleepiness was used. The sample included adolescents with and without ADHD given the documented rates of later evening preference, daytime sleepiness, and academic problems in adolescents with ADHD (Becker, Kapadia, Fershtman, & Sciberras, 2020; Langberg et al., 2016; Lunsford‐Avery, Krystal, & Kollins, 2016). This allowed us to evaluate whether the impact of circadian preference on daytime sleepiness and academic outcomes differed in an already at‐risk group. Furthermore, we controlled for a number of variables that have been associated with sleep problems and academic impairments, such as medication use (Lunsford‐Avery et al., 2016), sex (Arns et al., 2021), family income (Sivertsen, Bøe, Skogen, Petrie, & Hysing, 2017), and sleep duration (Becker, Epstein, et al., 2019).

To our knowledge, only one study has tested the prospective association between evening circadian preference and academic impairments and found no direct effects of evening preference (Scherrer & Preckel, 2021). Thus, we did not make specific hypothesis for the direct effect. Consistent with recent recommendations in the literature (Rucker, Preacher, Tormala, & Petty, 2011), our second objective was to test the indirect effect regardless of results for the direct effect of evening preference on academic outcomes. Specifically, researchers have raised concern with Baron and Kenny's (1986) criterion for establishing a direct effect before testing an indirect effect primarily related to the inadvertently omitting intervening effects (Mathieu & Taylor, 2006; Rucker et al., 2011). Given literature showing greater evening preference to be associated with increased ratings of daytime sleepiness (Luo et al., 2018; Roeser et al., 2013) and sleepiness to poor academic outcomes (Liu et al., 2016; Meijer, 2008), we anticipated an indirect effect of evening preference on academic outcomes via increased daytime sleepiness. Finally, we explored ADHD group status as a moderator of these indirect effects.

## Methods

### Participants

Participants were 302 adolescents (*n* = 162 with ADHD) between the ages of 12 and 14 years (*M* = 13.17; 44.7% females). Parents identified adolescents' race/ethnicity as European American/White (81.8%), multiracial (7.9%), Black/African American (5.3%), Asian (4.5%), and American Indian/Alaskan (0.3%). Slightly more than half of participants (53%) had a reported family income of $100,000 or higher, 31.2% between $50,000 and $100,000, and 14.5% less than $50,000. Further description of the sample and comparisons can be found elsewhere (Becker, Langberg, et al., 2019). Of the 302 participants, 288 completed rating scales at T2 (95.4% retention rate) and 266 completed rating scales at T3 (88% retention rate). Patterns of missingness are discussed in further detail next.

### Procedures

This study was approved by the institutional review board (IRB) at Cincinnati Children's Hospital Medical Center and Virginia Commonwealth University. This study uses data from three time points: the fall of 8th grade (T1), the fall of 9th grade (T2), and the fall/winter of 10th grade (T3). School staff at middle schools around Cincinnati, Ohio and Richmond, Virginia were provided with recruitment materials to distribute to families (letters/flyers to students, newsletters, e‐mail blasts). Parents contacted the research staff in response to recruitment materials and, after meeting general phone screening criteria (e.g., enrollment in eight grade), were invited for the initial visit. At this visit, the adolescent and parent received a comprehensive assessment, with inclusion criteria including: full scale IQ >80 on *Wechsler Abbreviated Scale of Intelligence, Second Edition* (WASI‐II) and meeting criteria for ADHD or comparison group. Adolescents eligible for the ADHD group met fifth edition of the *Diagnostic and Statistical Manual for Mental Disorders* (DSM‐5) criteria for ADHD combined presentation or inattentive presentation based on the parent version of the Children's Interview for Psychiatric Syndromes (P‐ChIPS; Weller, Weller, Teare, & Fristad, 1999). Adolescents were eligible for the comparison group if the parent endorsed <4 ADHD symptoms for both inattentive and hyperactive–impulsive domains. Exclusionary criteria included adolescents being previously diagnosed with or meeting criteria for autism spectrum disorder, bipolar disorder, dissociative disorder, a psychotic disorder, or an organic sleep disorder, or families who do not have sufficient English language to complete study materials. These exclusionary criteria resulted in 11 families being ineligible for the study. Parents provided written consent and adolescents provided assent at the first visit.

#### Diagnostic assessment

Adolescents were evaluated for ADHD at the initial visit in accordance with the DSM‐5 criteria. The parent version of the P‐ChIPS (Weller et al., 1999) was used to determine adolescent eligibility for the ADHD group. Adolescents were required to meet all DSM‐5 criteria for either ADHD combined presentation or predominately inattentive presentation according to parent report on the P‐ChIPS. Adolescents participated in the comparison group if parents endorsed ≤4 symptoms of both inattention and hyperactivity/impulsivity.

### Measures

#### Sleep habits survey (SHS)

The SHS (Wolfson & Carskadon, 1998) is a self‐report measure of circadian preference and sleep–wake difficulties. The 10‐item circadian preference subscale assesses preference for morningness or eveningness (‘When does your body start to tell you it is time for bed’?), with lower scores indicating greater evening preference. The circadian preference subscale has established internal consistency and convergent validity with ratings of later sleep onset and waketime (Giannotti et al., 2002; Russo, Bruni, Lucidi, Ferri, & Violani, 2007). Additionally, adolescents reported on their average sleep duration during the schoolweek. Internal consistency for circadian preference was α = .77.

#### Pediatric daytime sleepiness scale (PDSS)

The PDSS was adapted from the adult Epworth Sleepiness Scale for assessing adolescents' daytime sleepiness (Drake et al., 2003). Adolescents reported on their likelihood of falling asleep in different situations (e.g., while doing homework), extent of sleepiness or drowsiness (‘How often do you get sleepy or drowsy while doing homework?’), and difficulties waking on a 5‐point scale (0 = *never*, 4 = *very often*). Parents also provided their perception of adolescents' daytime sleepiness (‘How often does your child get sleepy or drowsy while doing homework?’). The PDSS has well‐documented psychometric properties and convergent validity with sleep problems (Drake et al., 2003; Liu et al., 2019). Internal consistencies for self and parent‐reported daytime sleepiness were αs = .84 and .82, respectively.

#### Impairment rating scale (IRS)

The IRS is a widely used rating scale of perceived impact of behaviors on domains of impairment (Fabiano et al., 2006). The IRS has demonstrated adequate test–retest reliability and convergent validity with measures of impairment across community and clinical samples (Fabiano et al., 2006). Parents (‘How your child's problems affect his or her academic progress at school’) and teacher (‘How this child's problems affect his or her academic progress’) are instructed to endorse on a 7‐point scale the extent of adolescents' problems in academics, with higher scores reflecting greater academic impairment and need for treatment in this area.

#### Student grades

School records for student grades in english/language arts, history, math, and science were obtained at the end of 8th grade and the end of 10th grade. Grades were quantified on a 4.0 grading scale consisting of a composite score of these subjects. Specific subject grades were also used in supplemental analyses.

#### Covariates

Parents were administered an adaptation of the Services Use in Children and Adolescents–Parent Interview (SCA‐PI; Hoagwood et al., 2004) to assess medication use (for ADHD, sleep [including melatonin], and/or an emotional/behavioral problem [e.g., antidepressants]). Medication use was coded as follows: 0 = no or unsure of medication status, 1 = yes. Parents also reported on family income, child's sex, and child's age.

### Analytic strategy

Significant demographic factors associated with T3 academic impairment ratings and GPA were used as covariates. Path analyses and tests of indirect effects were conducted in the statistical software *Mplus* (version 8.1). Manifest variables were used given the sample size, and all models were just identified (*df* = 0). Consistent with recommendations on handling nonignorable missing data, we used full‐information maximum likelihood with adolescent age and family income as auxiliary variables as an approach to reduce the impact of the nonignorable missing data biased parameter estimates (Graham, 2009; Nicholson, Deboeck, & Howard, 2017). We tested three indirect effect models, with T1 self‐reported circadian preference as the predictor, T2 self‐ or parent‐reported daytime sleepiness as the mediator, and T3 parent‐reported academic impairment, teacher‐reported academic impairment, and GPA as outcome variables in the separate models. We controlled for adolescent sex, age, ADHD group status, T1 medication status, T1 self‐reported sleep duration, and baseline academic impairment or GPA (in each model, respectively). The direct and indirect effects and their 95% confidence intervals (CIs) were calculated using 10,000 bias‐corrected bootstrapped sampling estimates (Mallinckrodt, Abraham, Wei, & Russell, 2006). Furthermore, aligning with recent recommendations on mediation (Rucker et al., 2011), we tested the indirect effect of these models even in the absence of a total or direct effect of T1 circadian preference on T3 academic impairment. Finally, as an exploratory analysis, we tested whether the indirect effect was moderated by ADHD group status (0 = comparison, 1 = ADHD).

## Results

### Participant attrition

There was a small‐to‐moderate amount of participant attrition across the three time points (7% for T2 daytime sleepiness measures; 12% for T3 parent‐reported academic impairment; 30% for teacher‐reported academic impairment). Furthermore, 3.6% of participants did not have GPA data at baseline, whereas 22.8% did not have GPA at the T3 visit. Little's MCAR test in SPSS indicated that data were missing in a pattern consistent with not missing at random (χ^2^(125) = 196.276, *p* < .001), with participants completing all time points being older, having a higher parent‐reported family income, and more likely to be in the non‐ADHD group (*p*s < .05). Thus, adolescent age and family income were used as auxiliary variables in missing data estimation moving forward. Primary study variables were normally distributed (skew <2.00, kurtosis <4.00).

### Bivariate associations

Table 1 presents zero‐order correlations and descriptive statistics between demographic variables and primary study variables. The following demographic factors were significantly correlated with T3 ratings of academic impairment and GPA: ADHD group status, older adolescents (only for academic impairment), adolescent girls (only for academic impairment), and families reporting lower household income (*p*s < .05). These variables were included as covariates in primary analyses, with age and family income as an auxiliary variable in missing data estimations.

**Table 1:** Means, standard deviations, and bivariate correlations of study variables

Variable	2	3	4	5	6	7	8	9	10	11	12	13	14	15	16	17	18	19	20	21	22	23
1. Group	−.02	−.21**	−.25**	.55**	−.07	−.10	.67**	.45**	−.49*	−.44**	−.45**	−.39**	−.41**	.09	.23**	.53**	.31**	−.36**	−.33**	−.27**	−.33**	−.32**
2. Age	–	−.09	.04	.01	−.05	−.14*	.07	.00	−.02	.03	−.07	−.01	−.01	.05	.07	.15*	.16*	−.07	−.06	−.04	−.06	−.05
3. Sex		–	−.02	−.06	−.13*	.02	−.20**	−.18**	.20**	.26**	.16**	.16**	.16**	.10	−.05	−.15*	−.25**	.09	.09	.02	.08	.14*
4. Income			–	−.22*	.12*	.11	−.27**	−.16*	.33**	.23**	.30**	.32**	.29**	−.12*	−.15*	−.20**	−.14*	.28**	.25**	.28**	.24**	.27**
5. Med				–	−.12*	−.10	.36**	.21**	−.25**	−.19**	−.23**	−.19**	−.25**	.10	.26**	.31**	.24**	−.20**	−.18**	−.17**	−.21**	−.15*
6. T1 duration					–	.20**	−.12*	−.06	.15**	.09	.09	.14*	.20*	−.17**	−.24**	−.08	−.12	.12	.08	.10	.05	.07
7. T1 preference						–	−.17**	−.08	.19**	.14*	.17**	.22**	.12*	−.32**	−.38**	−.20**	−.08	.11	.04	.00	.14*	.15*
8. T1 PR AI							–	.52**	−.58**	−.54**	−.52**	−.51**	−.46**	.09	.33**	.62**	.43**	−.44**	−.39**	−.33**	−.44**	−.35**
9. T1 TR AI								–	−.57**	−.54**	−.52**	−.48**	−.45**	.06	.11	.42**	.43**	−.40**	−.44**	−.28**	−.34**	−.33**
10. Baseline GPA									–	.88**	.88**	.88**	.85**	−.10	−.29**	−.52**	−.54**	.77**	.72**	.64**	.67**	.66**
11. Baseline English										–	.71**	.69**	.67**	−.05	−.22**	−.48**	−.48**	.66**	.64**	.52**	.55**	.59**
12. Baseline History											–	.67**	.65**	−.14*	−.22**	−.48**	−.46**	.65**	.63**	.55**	.54**	.56**
13. Baseline Math												–	.68**	−.08	−.28**	−.43**	−.50**	.72**	.63**	.60**	.66**	.62**
14. Baseline Science													–	−.06	−.27**	−.41**	−.44**	.63**	.59**	.54**	.59**	.49**
15. T2 SR sleepy														–	.43**	.19**	.11	−.09	−.06	−.07	−.08	−.08
16. T2 PR sleepy															–	.38**	.25**	−.28**	−.22**	−.25**	−.25**	−.25**
17. T3 PR AI																–	.54**	−.51**	−.46**	−.40**	−.41**	−.51**
18. T3 TR AI																	–	−58**	−.52**	−.51**	−.49**	−.52**
19. T3 GPA																		–	.88**	.87**	.87**	.87**
20. T3 English																			–	.69**	.65**	.69**
21. T3 History																				–	.68**	.68**
22. T3 Math																					–	.70**
23. T3 Science																						–

	1	2	3	4	5	6	7	8	9	10	11	12	13	14	15	16	17	18	19	20	21	22	23
*M*	*–*	–	–	–	–	7.9	26.89	2.01	1.52	3.12	3.10	3.10	3.01	3.22	1.57	1.54	1.71	1.30	3.05	3.07	3.11	3.01	3.02
*SD*	*–*	–	–	–	–	1.2	5.06	1.97	1.86	.74	.83	.91	.89	.79	.81	.67	1.90	1.76	.83	1.01	.92	.96	.88

For group, 0 = comparison, 1 = ADHD. For sex, 0 = male, 1 = female. T1 = time point 1 (fall 8th grade). T2 = time point 2 (fall 9th grade). T3 = time point 3 (middle of 10th grade). For Med, 0 = none, 1 = any medication. Duration = weekly average sleep duration. AI = academic impairment; Baseline, grades in the spring of 8th grade; Sleepy = daytime sleepiness; GPA = grade point average; PR = parent report; SR = adolescent self‐report; TR = teacher‐report.

*p < .05; **p < .01.

### Analyses testing the indirect effect of evening circadian preference on academic impairment via daytime sleepiness

The first set of analyses evaluated relations between T1 evening preference, T2 self or parent ratings of daytime sleepiness, and T3 parent‐reported academic impairment. Regarding effects of specific paths, T1 evening preference was uniquely associated with increased T3 parent‐reported academic impairment (β = −.117, *p* = .014), controlling for covariates and T1 parent‐reported academic impairment. ADHD status was associated with higher T3 ratings of academic impairment (β = −.184, *p* = .014). Additionally, T1 evening preference was uniquely associated with greater T2 self (β = −.335, *p* < .001) and parent (β = −.307, *p* < .0001) ratings of daytime sleepiness. Adolescent girls had greater T2 self‐reported daytime sleepiness (β = .122, *p* = .031), and T1 medication status (β = .152, *p* = .034), shorter sleep duration (β = −.142, *p* = .016), and parent‐reported academic impairment (β = .206, *p* = .004) were each significantly correlated with greater T2 parent‐reported daytime sleepiness. Finally, T2 self‐ (β = .101, *p* = .052) and parent‐reported daytime sleepiness (β = .147, *p* = .008) were uniquely associated with greater parent‐reported T3 academic impairment.

Second, we tested T1 evening preference, T2 self or parent ratings of daytime sleepiness, and T3 teacher‐reported academic impairment. Consistent with bivariate correlations, T1 evening preference was unrelated to T3 teacher‐reported academic impairments. Similar to the previous model with parent ratings of academic impairment, T1 evening preference was uniquely associated with both T2 self (β = −.338, *p* < .001) and parent (β = −.331, *p* < .001) ratings of daytime sleepiness. Similarly, T1 medication status (β = .152, *p* = .034) and shorter sleep duration (β = −.142, *p* = .016) were associated with greater T2 parent‐reported daytime sleepiness. T2 parent‐reported daytime sleepiness (β = .165, *p* = .030), but not self‐reported sleepiness (*p* > .05), were uniquely associated with increased T3 teacher‐reported academic impairment.

Finally, as presented in Table 2, we examined the indirect effect of T1 evening preference → T2 self‐ or parent‐reported daytime sleepiness → T3 academic impairment (for parent and teacher ratings, separately). The indirect effect of T1 evening preference → increased T3 parent‐reported academic impairment via greater T2 self and parent ratings of daytime sleepiness was significant. Furthermore, the indirect effect of T1 evening preference → increased T3 teacher‐reported academic impairment via greater T2 parent‐reported daytime sleepiness was significant. Conversely, the indirect effect via self‐reported sleepiness was nonsignificant (*p* > .05).

**Table 2:** Indirect effect results of T1 circadian preference, T2 self‐ and parent‐reported daytime sleepiness, and T3 parent‐ and teacher‐reported academic impairment

Indirect effect	Parent report of academic impairment				Teacher report of academic impairment			
	*ab*	*SE*	95% CI	*p*	*ab*	*SE*	95% CI	*p*
T2 SR sleepiness	−.044	.017	−.031, −.005	.012	−.034	.024	−.030, .005	.163
T2 PR sleepiness	−.052	.019	−.035, −.007	.006	−.062	.028	−.045, −.004	.027

All analyses also controlled for ADHD group status, sex, T1 medication status, T1 sleep duration, and baseline ratings of academic impairment. Standardized *ab* indirect effect and unstandardized confidence intervals are presented.

### Indirect effect of evening circadian preference on student grades via daytime sleepiness

Above and beyond covariates and baseline GPA (collected end of eighth grade), there was no direct effect of T1 evening preference on T3 GPA. Similar to the above models, T1 evening preference was uniquely associated with greater T2 self (β = −.333, *p* < .001) and parent (β = −.301, *p* < .001) ratings of daytime sleepiness. Furthermore, T2 parent‐reported daytime sleepiness (β = −.120, *p* = .020), but not self‐reported ratings of sleepiness (*p* > .05), was uniquely associated with lower T3 GPA, controlling for baseline covariates.

We then tested the indirect effect of T1 evening preference → T2 self‐ or parent‐reported daytime sleepiness → T3 GPA. As presented in Table 3, T1 evening preference was associated with lower T3 GPA through T2 parent‐reported daytime sleepiness (*ab* = .036, 95% CI [.001, .012], *p* = .032). The indirect effect of T1 evening preference to T3 GPA via T2 self‐reported daytime sleepiness was not significant (*p* > .05).

**Table 3:** Indirect effect results of T1 circadian preference, T2 self‐ and parent‐reported daytime sleepiness, and T3 school grades

Indirect effect	T3 student grades														
	Overall GPA			English			History			Math			Science		
	*ab*	95% CI	*p*	*ab*	95% CI	*p*	*ab*	95% CI	*p*	*ab*	95% CI	*p*	*ab*	95% CI	*p*
T2 SR sleepiness	−.012	−.003, .009	.454	.020	−.003, .012	.304	.009	−.005, .010	.643	.005	−.005, .008	.754	.012	−.005, .010	.549
T2 PR sleepiness	.036	.001, .012	.032	.041	.001, .017	.056	.058	.003, .021	.014	.026	−.001, .013	.153	.038	−.001, .016	.106

All analyses also controlled for ADHD group status, sex, T1 medication status, T1 sleep duration, and baseline grades. Standardized *ab* indirect effect and unstandardized confidence intervals are presented. GPA, grade point average; PR, parent report; SR, self‐report.

As supplemental analyses, we examined the direct and indirect effect on specific academic subjects. First, controlling for covariates and baseline grades, findings showed a direct effect of T1 evening preference on lower T3 science grades (β = −.140, *p* = .017), but not T3 english/language arts, history, or math grades (*p*s > .05). Furthermore, controlling for baseline covariates and grades, T2 parent‐reported daytime sleepiness was associated with lower T3 History (β = −.128, *p* = .033) and Science (β = −.150, *p* = .011) grades, but not english or math grades. T2 self‐reported daytime sleepiness was unrelated to T3 grades (*p*s > .05). Finally, as presented in Table 3, we then tested the indirect effect of T1 evening preference → T2 self‐ or parent‐reported daytime sleepiness → T3 grades. The indirect effects of T1 evening preference on lower T3 english and history grades through T2 parent‐reported daytime sleepiness were significant. None of the indirect effects via self‐reported daytime sleepiness were significant.

### Moderated indirect effect

Across all models, ADHD group status did not moderate the indirect effect of circadian preference on academic impairment, overall GPA, or specific academic subjects (*p*s > .05).^1^

## Discussion

The current study used a multi‐method (e.g., rating scale and GPA in core academic subjects obtained from school report cards), multi‐informant (e.g., self and parent ratings) design to test the prospective association of evening circadian preference, daytime sleepiness, and academic outcomes across 2 years in a sample of adolescents with and without ADHD. First, above and beyond demographic factors, ADHD group status, medication status, sleep duration, and baseline ratings of academic outcomes, factors shown in previous research to be associated with academic outcomes (Arns et al., 2021; Becker, Langberg, et al., 2019), adolescents' evening preference in the fall of 8th grade was uniquely associated with increased parent‐reported academic impairment in the middle/end of 10th grade. To our knowledge, these findings build on cross‐sectional studies (Saxvig et al., 2012; Sivertsen et al., 2015; Tonetti et al., 2015) by documenting prospective associations with ratings of academic impairment. With the exception of direct effects on lower science grades in 10th grade, evening preference was not directly related to teacher‐reported impairment, overall GPA, or other academic subject grades. These findings are consistent with the only longitudinal study to date finding morning circadian preference, but not evening preference (assessed on separate dimensions), to be directly associated with increases in overall GPA 2 years later (Scherrer & Preckel, 2021). However, consistent with recent recommendations for testing intervening mechanisms in the absence of direct effects to better understand developmental processes (Rucker et al., 2011), we sought to evaluate daytime sleepiness as a process that explain the indirect effect of evening preference on academic outcomes.

### Indirect effect via parent ratings of daytime sleepiness

In contrast to the limited direct effects, a consistent pattern emerged in which evening preference was indirectly associated with both parent and teacher ratings of academic impairments and lower overall GPA via parent ratings of daytime sleepiness. Importantly, these indirect effect findings were significant for adolescents' grades in 10th grade derived from school report cards, which is an important marker of academic achievement that strongly influences educational decision and success (Arbabi, Vollmer, Dörfler, & Randler, 2015; Scherrer & Preckel, 2021). These findings highlight the importance of considering mechanisms in studies examining how evening preference longitudinally influences functioning (Scherrer & Preckel, 2021). Adolescents with an evening preference experience a mismatch between sleep timing, optimal performance, and early school start times (Dolsen et al., 2019). In turn, the accompanying difficulties with morning waking and sleepiness during early portions of the school day likely have a cascading effect on academic performance. Indeed, experimental evidence demonstrates that adolescents with evening preferences perform poorly on cognitive measures earlier in the day (Goldstein, Hahn, Hasher, Wiprzycka, & Zelazo, 2007).

Interestingly, it was parent perceptions of adolescents' daytime sleepiness that consistently emerged as a mechanism of the link between evening preference and academic outcomes. Associations between parent ratings of adolescents' daytime sleepiness and academic impairment and grades were noticeably stronger compared to self‐reported ratings, consistent with a meta‐analyses finding larger effects of sleepiness on academic outcomes in studies using parent or objective measures of academic performance compared to self‐report (Dewald et al., 2010). Additionally, parents may be equipped to observe the impact of sleepiness on academic difficulties across contexts, such as observing heightened drowsiness upon morning waking and during homework completion (Owens, Dearth‐Wesley, Lewin, Gioia, & Whitaker, 2016).

Findings from supplemental analyses suggested that evening preference was directly associated with lower science grades and indirectly related to lower english and history grades via daytime sleepiness. Before interpreting these findings, we note that the significant direct effect of T1 evening preference on T3 math (per correlation results) and science (per correlation and direct effect results) grades were small in magnitude. Furthermore, findings from the direct and indirect effect of evening preference on specific grades were not consistent and findings should be interpreted cautiously. Still, a few studies have shown unique effects of sleep disturbances and sleepiness on verbal abilities and complex cognitive functions compared to nonverbal/automatic processes (Buckhalt, El‐Sheikh, & Keller, 2007; Macchitella, Marinelli, Signore, Ciavolino, & Angelelli, 2020), whereas other studies find elevated sleepiness to undermine working memory and processing speed abilities (Calhoun et al., 2012) and impact math performance (Macchitella et al., 2020). Our findings for specific subject areas need to be replicated before drawing conclusions, and additional prospective studies evaluating circadian preference on specific academic subjects are needed to shed light on possible underlying neurocognitive or learning mechanisms.

Finally, the magnitude of indirect effects did not differ for adolescents with and without ADHD. Although surprising at first given documented elevations in evening preference, daytime sleepiness, and academic impairments in adolescents with ADHD (Langberg et al., 2016; Lunsford‐Avery et al., 2016), these findings simply mean that evening preference and daytime sleepiness do not differentially or disproportionately impact the academic outcomes of adolescents with ADHD. Indeed, studies have found evening preference to be associated with daytime sleepiness and academic impairments across general community‐based samples of adolescents (Luo et al., 2018; Roeser et al., 2013; Scherrer & Preckel, 2021).

Collectively, our findings highlight the role of daytime sleepiness as an intervening mechanism linking circadian evening preference with academic outcomes. With few exceptions, these findings indicate that evening preference and academic outcomes may not be related directly but through their relationship with daytime sleepiness (Mathieu & Taylor, 2006). Moving forward, as research continues to investigate circadian function and daytime sleepiness on academic adjustment, it will be critical for longitudinal studies to explore *specific* underlying neurocognitive and academic processes underlying this developmental sequence. Regarding the synchrony of circadian preference and peak performance (Goldstein et al., 2007), increased daytime sleepiness may result in disruptions on attention regulation, learning or motivation, homework completion, or specific neurocognitive processes (e.g., verbal comprehension, processing speed; Buckhalt et al., 2007; Sivertsen et al., 2015). Future research, potentially using ecological momentary assessment or daily diary studies, would be well equipped to elucidate whether the impact is limited to the morning period or cascades into later periods and classes during the school day.

### Limitations

Despite a number of strengths, including a multi‐informant examination of the longitudinal association of circadian preference, daytime sleepiness, and academic impairment over 2 years in adolescents with and without ADHD, a few limitations are important to note. First, our prospective observational study precludes drawing causal influences of evening preference on sleepiness and academic performance. Thus, we encourage future research to consider implementing experimental designs to better understand mechanisms linking evening preference to increased sleepiness and, in turn, poor academic outcomes. Specifically, similar to studies examining effects on cognitive abilities (Goldstein et al., 2007), randomly assigning adolescents with late circadian preference to complete academic work at different times of the day or at times when circadian rhythm aligns with peak (or poor) performance. Second, our measure of evening circadian preference is not a direct measure of circadian function, and future research should include biological indices of circadian functioning such as dim light melatonin onset (Zavada et al., 2005). Importantly, objective measures of chronotype often find intraindividual variance across adolescence (Karan et al., 2021), pointing to the importance of repeated assessments and to better understand if evening preference stability and change is associated with sleepiness and academic outcomes. Along these lines, subjective ratings of daytime sleepiness introduce risk of bias, and future research is encouraged to consider objective or naturalistic designs to capture daytime sleepiness and drowsiness. Second, we did not have information related to school start times and whether this moderated indirect effects, which is a key area for future research. School timing has been found to influence the association between preference and academic performance (Goldin, Sigman, Braier, Golombek, & Leone, 2020), though contradictory findings have been reported as well (Martin, Gaudreault, Perron, & Laberge, 2016). Finally, participants who completed ratings across the three time points had higher family income and were more likely to be in the non‐ADHD group; thus, future research is encouraged to include adolescents of diverse socioeconomic and racial backgrounds to further understand the effect of evening preference on sleepiness and academic outcomes.

## Conclusion

Findings from the current study are the first to identify daytime sleepiness as a mechanism linking evening circadian preference and academic outcomes across a 2‐year period in adolescence. Specifically, self‐reported evening circadian preference was associated with increased parent ratings of academic impairment through self‐ and parent‐reported daytime sleepiness, whereas parent ratings of daytime sleepiness explained the link between evening preference and increased teacher‐reported impairment and lower GPA, in addition to lower grades in english/language arts and history subjects specifically. These findings highlight the continued need for longitudinal mediation studies to better understand how circadian factors impact academic adjustment in adolescents.

## Notes

1 Given differences in type of T1 medication status (31% ADHD medication; 12% sleep medication; 9% medication for emotional/behavioral problems), we decided to re‐run analyses with each medication type as a covariate separately. Results for the indirect effect and moderated indirect effect were unchanged.

## References

[jcpp13683-bib-0001] Arbabi, T. , Vollmer, C. , Dörfler, T. , & Randler, C. (2015). The influence of chronotype and intelligence on academic achievement in primary school is mediated by conscientiousness, midpoint of sleep and motivation. Chronobiology International, 32, 349–357. 25392281 10.3109/07420528.2014.980508

[jcpp13683-bib-0002] Arns, M. , Kooij, J.J.S. , & Coogan, A.N. (2021). Review: Identification and management of circadian rhythm sleep disorders as a transdiagnostic feature in child and adolescent psychiatry. Journal of the American Academy of Child & Adolescent Psychiatry, 60, 1085–1095. 33556454 10.1016/j.jaac.2020.12.035

[jcpp13683-bib-0003] Becker, S.P. , Epstein, J.N. , Tamm, L. , Tilford, A.A. , Tischner, C.M. , Isaacson, P.A. `, … & Beebe, D.W. (2019). Shortened sleep duration causes sleepiness, inattention, and oppositionality in adolescents with attention‐deficit/hyperactivity disorder: Findings from a crossover sleep restriction/extension study. Journal of the American Academy of Child & Adolescent Psychiatry, 58, 433–442. 30768404 10.1016/j.jaac.2018.09.439 PMC6441371

[jcpp13683-bib-0004] Becker, S.P. , Kapadia, D.K. , Fershtman, C.E. , & Sciberras, E. (2020). Evening circadian preference is associated with sleep problems and daytime sleepiness in adolescents with ADHD. Journal of Sleep Research, 29, e12936. 31651076 10.1111/jsr.12936 PMC6944746

[jcpp13683-bib-0005] Becker, S.P. , Langberg, J.M. , & Byars, K.C. (2015). Advancing a biopsychosocial and contextual model of sleep in adolescence: A review and introduction to the special issue. Journal of Youth and Adolescence, 44, 239–270. 25552436 10.1007/s10964-014-0248-y

[jcpp13683-bib-0006] Becker, S.P. , Langberg, J.M. , Eadeh, H.M. , Isaacson, P.A. , & Bourchtein, E. (2019). Sleep and daytime sleepiness in adolescents with and without ADHD: Differences across ratings, daily diary, and actigraphy. Journal of Child Psychology and Psychiatry, 60, 1021–1031. 31032953 10.1111/jcpp.13061 PMC6692210

[jcpp13683-bib-0007] Buckhalt, J.A. , El‐Sheikh, M. , & Keller, P. (2007). Children's sleep and cognitive functioning: Race and socioeconomic status as moderators of effects. Child Development, 78, 213–231. 17328701 10.1111/j.1467-8624.2007.00993.x

[jcpp13683-bib-0008] Calhoun, S.L. , Fernandez‐Mendoza, J. , Vgontzas, A.N. , Mayes, S.D. , Tsaoussoglou, M. , Rodriguez‐Muñoz, A. , & Bixler, E.O. (2012). Learning, attention/hyperactivity, and conduct problems as sequelae of excessive daytime sleepiness in a general population study of young children. Sleep, 35, 627–632. 22547888 10.5665/sleep.1818 PMC3321421

[jcpp13683-bib-0009] Chen, S.‐J. , Zhang, J.‐H. , Li, S.X. , Tsang, C.C. , Chan, K.C.C. , Au, C.T. , … & Chan, N.Y. (2021). The trajectories and associations of eveningness and insomnia with daytime sleepiness, depression and suicidal ideation in adolescents: A 3‐year longitudinal study. Journal of Affective Disorders, 294, 533–542. 34330050 10.1016/j.jad.2021.07.033

[jcpp13683-bib-0010] Chung, K.‐F. , & Cheung, M.‐M. (2008). Sleep‐wake patterns and sleep disturbance among Hong Kong Chinese adolescents. Sleep, 31, 185–194. 18274265 10.1093/sleep/31.2.185 PMC2225574

[jcpp13683-bib-0011] Chung, S.J. , An, H. , & Suh, S. (2020). What do people do before going to bed? A study of bedtime procrastination using time use surveys. Sleep, 43, zsz267. 31680171 10.1093/sleep/zsz267

[jcpp13683-bib-0012] Cohen‐Zion, M. , & Shiloh, E. (2018). Evening chronotype and sleepiness predict impairment in executive abilities and academic performance of adolescents. Chronobiology International, 35, 137–145. 29111789 10.1080/07420528.2017.1387792

[jcpp13683-bib-0013] Crowley, S.J. , Wolfson, A.R. , Tarokh, L. , & Carskadon, M.A. (2018). An update on adolescent sleep: New evidence informing the perfect storm model. Journal of Adolescence, 67, 55–65. 29908393 10.1016/j.adolescence.2018.06.001 PMC6054480

[jcpp13683-bib-0014] Dewald, J.F. , Meijer, A.M. , Oort, F.J. , Kerkhof, G.A. , & Bögels, S.M. (2010). The influence of sleep quality, sleep duration and sleepiness on school performance in children and adolescents: A meta‐analytic review. Sleep Medicine Reviews, 14, 179–189. 20093054 10.1016/j.smrv.2009.10.004

[jcpp13683-bib-0015] Dolsen, M.R. , Wyatt, J.K. , & Harvey, A.G. (2019). Sleep, circadian rhythms, and risk across health domains in adolescents with an evening circadian preference. Journal of Clinical Child & Adolescent Psychology, 48, 480–490. 29368957 10.1080/15374416.2017.1416620 PMC6060007

[jcpp13683-bib-0016] Drake, C. , Nickel, C. , Burduvali, E. , Roth, T. , Jefferson, C. , & Badia, P. (2003). The pediatric daytime sleepiness scale (PDSS): Sleep habits and school outcomes in middle‐school children. Sleep, 26, 455–458. 12841372

[jcpp13683-bib-0017] Fabiano, G.A. , Pelham, J. , William, E. , Waschbusch, D.A. , Gnagy, E.M. , Lahey, B.B. , … & Burrows‐MacLean, L. (2006). A practical measure of impairment: Psychometric properties of the impairment rating scale in samples of children with attention deficit hyperactivity disorder and two school‐based samples. Journal of Clinical Child and Adolescent Psychology, 35, 369–385. 16836475 10.1207/s15374424jccp3503_3

[jcpp13683-bib-0018] Giannotti, F. , Cortesi, F. , Sebastiani, T. , & Ottaviano, S. (2002). Circadian preference, sleep and daytime behaviour in adolescence. Journal of Sleep Research, 11, 191–199. 12220314 10.1046/j.1365-2869.2002.00302.x

[jcpp13683-bib-0019] Goldin, A.P. , Sigman, M. , Braier, G. , Golombek, D.A. , & Leone, M.J. (2020). Interplay of chronotype and school timing predicts school performance. Nature Human Behaviour, 4, 387–396. 10.1038/s41562-020-0820-2 32042108

[jcpp13683-bib-0020] Goldstein, D. , Hahn, C.S. , Hasher, L. , Wiprzycka, U.J. , & Zelazo, P.D. (2007). Time of day, intellectual performance, and behavioral problems in morning versus evening type adolescents: Is there a synchrony effect? Personality and Individual Differences, 42, 431–440. 17268574 10.1016/j.paid.2006.07.008 PMC1761650

[jcpp13683-bib-0021] Graham, J.W. (2009). Missing data analysis: Making it work in the real world. Annual Review of Psychology, 60, 549–576. 10.1146/annurev.psych.58.110405.085530 18652544

[jcpp13683-bib-0022] Hoagwood, K. , Jensen, P.S. , Arnold, L.E. , Roper, M. , Severe, J. , Odbert, C. , … Group, M. T. A. C . (2004). Reliability of the services for children and adolescents‐parent interview. Journal of the American Academy of Child and Adolescent Psychiatry, 43, 1345–1354. 15502593 10.1097/01.chi.0000139558.54948.1f

[jcpp13683-bib-0023] Jenni, O.G. , Achermann, P. , & Carskadon, M.A. (2005). Homeostatic sleep regulation in adolescents. Sleep, 28, 1446–1454. 16335485 10.1093/sleep/28.11.1446

[jcpp13683-bib-0024] Karan, M. , Bai, S. , Almeida, D.M. , Irwin, M.R. , McCreath, H. , & Fuligni, A.J. (2021). Sleep–wake timings in adolescence: Chronotype development and associations with adjustment. Journal of Youth and Adolescence, 50(4), 628–640. 33606125 10.1007/s10964-021-01407-1 PMC7993411

[jcpp13683-bib-0025] Kuula, L. , Pesonen, A.‐K. , Merikanto, I. , Gradisar, M. , Lahti, J. , Heinonen, K. , … & Räikkönen, K. (2018). Development of late circadian preference: Sleep timing from childhood to late adolescence. The Journal of Pediatrics, 194, 182–189.e1. 29221693 10.1016/j.jpeds.2017.10.068

[jcpp13683-bib-0026] Langberg, J.M. , Dvorsky, M.R. , Molitor, S.J. , Bourchtein, E. , Eddy, L.D. , Smith, Z. , … & Evans, S.W. (2016). Longitudinal evaluation of the importance of homework assignment completion for the academic performance of middle school students with ADHD. Journal of School Psychology, 55, 27–38. 26931065 10.1016/j.jsp.2015.12.004 PMC5134299

[jcpp13683-bib-0027] Liu, J. , Liu, X. , Ji, X. , Wang, Y. , Zhou, G. , & Chen, X. (2016). Sleep disordered breathing symptoms and daytime sleepiness are associated with emotional problems and poor school performance in children. Psychiatry Research, 242, 218–225. 27289327 10.1016/j.psychres.2016.05.017 PMC4976000

[jcpp13683-bib-0028] Liu, Y. , Zhang, J. , Li, S.X. , Chan, N.Y. , Yu, M.W.M. , Lam, S.P. , … & Wing, Y.K. (2019). Excessive daytime sleepiness among children and adolescents: Prevalence, correlates, and pubertal effects. Sleep Medicine, 53, 1–8. 30384136 10.1016/j.sleep.2018.08.028

[jcpp13683-bib-0029] Lunsford‐Avery, J.R. , Krystal, A.D. , & Kollins, S.H. (2016). Sleep disturbances in adolescents with ADHD: A systematic review and framework for future research. Clinical Psychology Review, 50, 159–174. 27969004 10.1016/j.cpr.2016.10.004 PMC5401800

[jcpp13683-bib-0030] Luo, C. , Zhang, J. , Chen, W. , Lu, W. , & Pan, J. (2018). Course, risk factors, and mental health outcomes of excessive daytime sleepiness in rural Chinese adolescents: A one‐year prospective study. Journal of Affective Disorders, 231, 15–20. 29408158 10.1016/j.jad.2018.01.016

[jcpp13683-bib-0031] Macchitella, L. , Marinelli, C.V. , Signore, F. , Ciavolino, E. , & Angelelli, P. (2020). Sleepiness, neuropsychological skills, and scholastic learning in children. Brain Sciences, 10, 529. 32784660 10.3390/brainsci10080529 PMC7464965

[jcpp13683-bib-0032] Mallinckrodt, B. , Abraham, W.T. , Wei, M. , & Russell, D.W. (2006). Advances in testing the statistical significance of mediation effects. Journal of Counseling Psychology, 53, 372–378.

[jcpp13683-bib-0033] Martin, J.S. , Gaudreault, M.M. , Perron, M. , & Laberge, L. (2016). Chronotype, light exposure, sleep, and daytime functioning in high school students attending morning or afternoon school shifts. Journal of Biological Rhythms, 31, 205–217. 26825618 10.1177/0748730415625510

[jcpp13683-bib-0034] Mathieu, J.E. , & Taylor, S.R. (2006). Clarifying conditions and decision points for mediational type inferences in organizational behavior. Journal of Organizational Behavior, 27, 1031–1056.

[jcpp13683-bib-0035] Meijer, A.M. (2008). Chronic sleep reduction, functioning at school and school achievement in preadolescents. Journal of Sleep Research, 17, 395–405. 19021856 10.1111/j.1365-2869.2008.00677.x

[jcpp13683-bib-0036] Nicholson, J.S. , Deboeck, P.R. , & Howard, W. (2017). Attrition in developmental psychology: A review of modern missing data reporting and practices. International Journal of Behavioral Development, 41, 143–153.

[jcpp13683-bib-0037] Owens, J.A. , Dearth‐Wesley, T. , Lewin, D. , Gioia, G. , & Whitaker, R.C. (2016). Self‐regulation and sleep duration, sleepiness, and chronotype in adolescents. Pediatrics, 138, e20161406. 27940688 10.1542/peds.2016-1406

[jcpp13683-bib-0038] Roeser, K. , Schlarb, A.A. , & Kübler, A. (2013). The Chronotype‐Academic Performance Model (CAM): Daytime sleepiness and learning motivation link chronotype and school performance in adolescents. Personality and Individual Differences, 54, 836–840.

[jcpp13683-bib-0039] Rucker, D.D. , Preacher, K.J. , Tormala, Z.L. , & Petty, R.E. (2011). Mediation analysis in social psychology: Current practices and new recommendations. Social and Personality Psychology Compass, 5, 359–371.

[jcpp13683-bib-0040] Russo, P.M. , Bruni, O. , Lucidi, F. , Ferri, R. , & Violani, C. (2007). Sleep habits and circadian preference in Italian children and adolescents. Journal of Sleep Research, 16, 163–169. 17542946 10.1111/j.1365-2869.2007.00584.x

[jcpp13683-bib-0041] Saxvig, I.W. , Pallesen, S. , Wilhelmsen‐Langeland, A. , Molde, H. , & Bjorvatn, B. (2012). Prevalence and correlates of delayed sleep phase in high school students. Sleep Medicine, 13, 193–199. 22153780 10.1016/j.sleep.2011.10.024

[jcpp13683-bib-0042] Scherrer, V. , & Preckel, F. (2021). Circadian preference and academic achievement in school‐aged students: A systematic review and a longitudinal investigation of reciprocal relations. Chronobiology International, 38, 1195–1214. 33980088 10.1080/07420528.2021.1921788

[jcpp13683-bib-0043] Sivertsen, B. , Bøe, T. , Skogen, J.C. , Petrie, K.J. , & Hysing, M. (2017). Moving into poverty during childhood is associated with later sleep problems. Sleep Medicine, 37, 54–59. 28899540 10.1016/j.sleep.2017.06.005

[jcpp13683-bib-0044] Sivertsen, B. , Glozier, N. , Harvey, A.G. , & Hysing, M. (2015). Academic performance in adolescents with delayed sleep phase. Sleep Medicine, 16, 1084–1090. 26298783 10.1016/j.sleep.2015.04.011

[jcpp13683-bib-0045] Tonetti, L. , Natale, V. , & Randler, C. (2015). Association between circadian preference and academic achievement: A systematic review and meta‐analysis. Chronobiology International, 32, 792–801. 26125131 10.3109/07420528.2015.1049271

[jcpp13683-bib-0046] Vollmer, C. , Jankowski, K.S. , Díaz‐Morales, J.F. , Itzek‐Greulich, H. , Wüst‐Ackermann, P. , & Randler, C. (2017). Morningness–eveningness correlates with sleep time, quality, and hygiene in secondary school students: A multilevel analysis. Sleep Medicine, 30, 151–159. 28215240 10.1016/j.sleep.2016.09.022

[jcpp13683-bib-0047] Weller, E. , Weller, R. , Teare, M. , & Fristad, M. (1999). Parent version‐children's interview for psychiatric syndromes (P‐ChIPS). Washington, DC: American Psychiatric.

[jcpp13683-bib-0048] Wolfson, A.R. , & Carskadon, M.A. (1998). Sleep schedules and daytime functioning in adolescents. Child Development, 69, 875–887. 9768476

[jcpp13683-bib-0049] Zavada, A. , Gordijn, M.C. , Beersma, D.G. , Daan, S. , & Roenneberg, T. (2005). Comparison of the Munich Chronotype Questionnaire with the Horne‐Östberg's morningness‐eveningness score. Chronobiology International, 22, 267–278. 16021843 10.1081/cbi-200053536

